# Biotic and Abiotic Factors Influencing Arsenic Biogeochemistry and Toxicity in Fluvial Ecosystems: A Review

**DOI:** 10.3390/ijerph17072331

**Published:** 2020-03-30

**Authors:** Laura Barral-Fraga, María Teresa Barral, Keeley L. MacNeill, Diego Martiñá-Prieto, Soizic Morin, María Carolina Rodríguez-Castro, Baigal-Amar Tuulaikhuu, Helena Guasch

**Affiliations:** 1 Grup de recerca en Ecologia aquàtica continental (GRECO), Departament de Ciències Ambientals, Universitat de Girona, 17071 Girona, Spain; helena.guasch@ceab.csic.es; 2 LDAR24—Laboratoire Départemental d’Analyse et de Recherche du Département de la Dordogne, 24660 Coulounieix-Chamiers, Périgueux, France; 3 Instituto CRETUS, Departmento de Edafoloxía e Química Agrícola, Facultade de Farmacia, Campus Vida, Universidade de Santiago de Compostela, 15782 Santiago de Compostela, Spain; mteresa.barral@usc.es (M.T.B.); diegomartinha@gmail.com (D.M.-P.); 4 Forest Ecosystems and Society, Oregon State University, Corvallis, OR 97331, USA; keeleymacneill@gmail.com; 5 INRAE—Institut National de Recherche en Agriculture, Alimentation et Environnement, UR EABX—Equipe ECOVEA, 33612 Cestas Cedex, France; soizic.morin@inrae.fr; 6 INEDES—Instituto de Ecología y Desarrollo Sustentable (UNLu-CONICET), Universidad Nacional de Luján, 6700 Buenos Aires, Argentina; carolina.rodriguez.castro@gmail.com; 7 CONICET—Consejo Nacional de Investigaciones Científicas y Técnicas, Ciudad Autónoma de Buenos Aires C1425FQB CABA, Argentina; 8 School of Agroecology, Mongolian University of Life Sciences, Khoroo 11, Ulaanbaatar 17024, Mongolia; tbaigalamar@muls.edu.mn; 9 CEAB—Centre d’Estudis Avançats de Blanes, CSIC, Blanes, 17300 Girona, Spain

**Keywords:** arsenic, biofilm, microalgae, bacteria, phosphate, biogeochemistry, speciation, toxicity, ecotoxicology, trophic interactions

## Abstract

This review is focused on the biogeochemistry of arsenic in freshwaters and, especially, on the key role that benthic microalgae and prokaryotic communities from biofilms play together in through speciation, distribution, and cycling. These microorganisms incorporate the dominant iAs (inorganic arsenic) form and may transform it to other arsenic forms through metabolic or detoxifying processes. These transformations have a big impact on the environmental behavior of arsenic because different chemical forms exhibit differences in mobility and toxicity. Moreover, exposure to toxicants may alter the physiology and structure of biofilms, leading to changes in ecosystem function and trophic relations. In this review we also explain how microorganisms (i.e., biofilms) can influence the effects of arsenic exposure on other key constituents of aquatic ecosystems such as fish. At the end, we present two real cases of fluvial systems with different origins of arsenic exposure (natural vs. anthropogenic) that have improved our comprehension of arsenic biogeochemistry and toxicity in freshwaters, the Pampean streams (Argentina) and the Anllóns River (Galicia, Spain). We finish with a briefly discussion of what we consider as future research needs on this topic. This work especially contributes to the general understanding of biofilms influencing arsenic biogeochemistry and highlights the strong impact of nutrient availability on arsenic toxicity for freshwater (micro) organisms.

## 1. Arsenic Occurrence and Fate in Freshwater Environments

Arsenic (As) is widely dispersed in the Earth’s crust (e.g., [1]), where it ranks as the 20th most abundant trace element [2]. It is also found in soil, freshwater, and marine environments, with no known biological benefit (e.g., [3]). In some environments, arsenic is considered a serious environmental issue (e.g., [4]) because of its high concentration and harmful effects on organisms, directly by absorption or indirectly through the food chain pathways (e.g., [5]). Arsenic contamination of natural waters (groundwater, seawater, and freshwater) is a worldwide problem as high concentrations have been reported for water samples in several parts of the world [6,7,8], and over 200 million persons globally are at risk of arsenic exposure at levels of concern for human health [9]. The United States Environmental Protection Agency (U.S. EPA) sets limits for arsenic concentration in freshwaters to protect aquatic organisms from arsenic. It has established criteria maximum concentrations (CMC) and criteria continuous concentrations (CCC) for most inland surface waters. The CMC is the highest concentration of a contaminant in a surface water that will not show any deleterious effects to aquatic life after exposure for a brief period of time (an average of 1 h), while the CCC is the highest concentration of a contaminant for which aquatic life will not show any deleterious effects after an extended period of time [10] (after exposure for several days). According to the U.S. EPA, the CMC for acute arsenic exposure is 340 μg As L^−1^, while the CCC for chronic arsenic exposure is 150 μg As L^−1^ [11]. A much lower concentration (10 µg As L^−1^) was established as the maximum concentration limit for drinking water by the U.S. EPA, the European Union (EU) Council Directive, and the World Health Organization [12,13,14], and drinking water companies aim to supply drinking water with <1 μg As L^−1^ [15]. Therefore, arsenic exposure thresholds for human health are 15 times lower than those established for environmental health.

## 2. Arsenic Sources 

**Figure 1:**
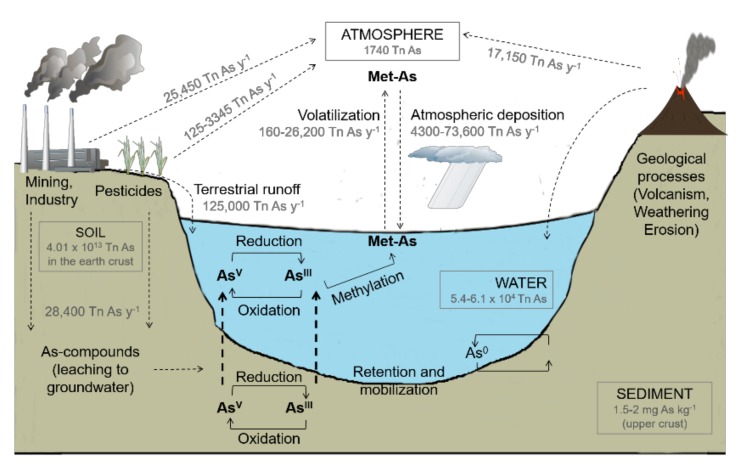
Global arsenic (As) biogeocycle (modified from [17], after [16]), with some estimated arsenic fluxes from [23]. Met-As= methylarsenicals; As^V^ = arsenate; As^III^ = arsenite; As^0^ = arsenic; t = tons; t y^−1^ = tons per year.

Arsenic enters the atmosphere through dust particles coming from volcanic emissions (ashes), wind erosion, low-temperature volatilization from soils, marine aerosols, and pollution and is returned to the Earth’s surface (mainly to water bodies) by atmospheric deposition; then, it moves through terrestrial runoff and groundwater discharge to the water bodies (Figure 1). There, it binds to or (co)precipitates with suspended particles and tends to sink to the sediments (e.g., [16,17]).

Arsenic is a constituent of more than 200 minerals (e.g., [1]) and is primarily present in chemically reduced forms, like realgar (AsS), orpiment (As_2_S_3_), and arsenopyrite (FeAsS), the latter being the most abundant arsenic ore (e.g., [7]). Natural geological sources are some of the most significant causes of arsenic contaminated groundwaters around the world [18,19,20,21,22]. 

Arsenic contamination is frequently observed in soils, sediments, and water. While baseline arsenic concentration is on average below 1 μg L^−1^, values above 10 μg L^−1^ are commonly reported in polluted European rivers (e.g., [6,7]). High arsenic concentrations in surface waters is in most cases attributed to the discharge of groundwaters (in cases with values up to 21,800 μg As L^−1^), mining activities (values can reach 7900 μg As L^−1^), or the influence of geothermal activity (values up to 370 μg As L^−1^), as reviewed by Smedley and Kinniburgh (2002) [7]. Arsenic can be mobilized in gold mining extractions, due to its co-occurrence with gold (e.g., [1]), and through other arsenic anthropogenic inputs, including indiscriminate use of certain pesticides and herbicides, as well as of wood preserving arsenicals [1,5]. 

## 3. Arsenic Speciation in Freshwater Ecosystems

Arsenic may occur in the environment in four oxidation states: +V (arsenate), +III (arsenite), 0 (arsenic), and -III (arsine). In natural waters, it is mostly found in the inorganic form (iAs) and as oxyanions of pentavalent arsenate (As^V^) and of trivalent arsenite (As^III^) [8,19,24]. The quantification of arsenic species in water may be a difficult task since changes in the relative proportion of arsenic species may occur rapidly after sampling. For example, arsenite is so easily oxidized to arsenate (especially by microorganisms, since they enhance this process at least seven orders of magnitude faster than the abiotic rate [25]) that a lack of an immediate and appropriate method to preserve species could result in questionable speciation data [26,27,28]. Concentrations and relative proportions of arsenic species vary according to changes in arsenic source, redox conditions, and biological activity (e.g., [7]). Usually, arsenate is the thermodynamically stable state in oxic waters, while arsenite is predominant in anoxic and reduced environments [7,8]. Consequently, in oxic waters of lakes, rivers, and oceans, arsenate is generally the dominant species, whereas high relative proportions of arsenite have been found in groundwater, in the hypolimnion of lakes with anoxic bottom waters, in river stretches close to inputs of arsenite-dominated industrial effluents, and in waters with a component of geothermal water [6,7].

Redox potential (Eh) and pH are generally considered the most important factors controlling arsenic speciation (e.g., [6]), but the presence of arsenite can be maintained in oxic waters by biological reduction of arsenate (e.g., [7]). In fact, autotrophic and heterotrophic communities play a key role in arsenic biogeochemistry (speciation, distribution, and cycling) in aquatic systems, since they incorporate the dominant iAs and may convert it to other arsenic forms such as the organic methylarsenicals (Met-As) and/or organoarsenicals (orgAs) like arsenosugars (e.g., [8,24]). Their biological activities may affect the speciation and bioavailability of arsenic in water and sediments (e.g., [29]), participating actively in the arsenic environmental cycle (e.g., [30]). These reactions have a big impact on the environmental behavior of arsenic, since the different chemical forms of this element exhibit different abilities to be mobilized or distributed in the environment (methyl-As^III^ >> methyl-As^V^ > As^III^ > As^V^) and toxicity to higher organisms (methyl-As^III^ > As^III^ > As^V^ > methyl-As^V^) (e.g., [31]). 

## 4. Arsenic in Sediments and Sediment-Water Interactions

Sediments are considered the ultimate sink for many pollutants in the aquatic environment (e.g., [32]). In freshwater systems, arsenic is predominantly bound to sediments, which may show high concentrations of this element (e.g., [33]). Particularly in mining areas, the concentration of arsenic can reach up to thousands of mg kg^−1^ sediment (e.g., [7]). Arsenic may be incorporated into sediments as arsenic-enriched particles eroded from weathered rock or soil and as colloids carrying adsorbed arsenic, which settle under low energy conditions. It can also reach stream beds as soluble arsenic that is retained by the inorganic, organic, and biotic components of sediments (e.g., [19]).

Sediments can act as sinks and as sources of contaminants in aquatic systems (e.g., [34]). Although they play an important role in maintaining water quality by removing contaminants from the water column, subsequent remobilization can lead to increased concentrations long after the cause of the contamination has ceased (e.g., [35]). Arsenic remobilization is particularly important to consider; although freshwater sediments usually act as sinks for this element in river systems, changes in environmental conditions (Eh, pH, water composition, physical disturbance) may promote arsenic release from this compartment to the water. In this case, arsenic enriched sediments may act as ‘‘chemical time bombs’’ (e.g., [36]), as they may release arsenic under certain favorable circumstances, causing a risk to aquatic life and human health [37,38].

Arsenic distribution between the water column and the sediment is controlled by several physico-chemical and biological processes, such as precipitation/solubilization, adsorption/desorption, oxidation/reduction, incorporation in the crystal structure of minerals, and biological exchanges [39,40]. The first three mechanisms are the most relevant among those of a physical-chemical nature and will be explained in the following subsections. Transformations in arsenic speciation, mobilization, and toxicity caused directly by microorganisms will be explained later on. 

### 4.1. Precipitation/Dissolution

Some conditions can cause arsenate and arsenite to precipitate out of solution. The topic has been extensively described by Smedley and Kinniburgh (2002), Mandal and Suzuki (2002), Drahota and Filippi (2009) [7,41,42]. Arsenate, like phosphate (PO_4_^3^^−^), can precipitate with aluminum and ferric iron under low pH conditions and with calcium and magnesium under high pH conditions (e.g., [43]). Arsenate may also replace sulfate (SO_4_^2−^) or phosphate in minerals, due to the similar stereochemistry and charge (e.g., [7]). In contrast, arsenite can precipitate with sulfide, thus decreasing soluble arsenite concentration in very reducing environments [44,45]. Solubilization by mineral dissolution is mainly due to reductive dissolution of Fe and Mn (hydr) oxides carrying coprecipitated or adsorbed arsenic.

### 4.2. Oxidation/Reduction

The oxidation reduction potential (Eh) plays an important role in arsenic mobility, as it affects the affinity of arsenite and arsenate species for the various sorbents; e.g., it has been demonstrated that arsenite forms more labile complexes with iron oxide surfaces [46]. Furthermore, various studies have demonstrated the release of arsenic under reducing conditions in the sediments [44,47,48,49]. Iron reduction and dissolution have been associated with the release of arsenic [50,51], but arsenic reduction may also be influential.

### 4.3. Adsorption/Desorption

Adsorption, that is enrichment of one or more components in an interfacial layer [52], is considered the main mechanism for arsenic retention in freshwater sediments and the cause of the low concentrations of arsenic observed in most natural waters [7,53,54,55,56,57], although adsorbed As remains still relatively bioavailable compared to (co)precipitated/incorporated arsenic. Arsenic adsorption capacity has been related to both the content of metal oxides, particularly of Fe, Al, and Mn [7,51,58], and to the clay content of the sediments (e.g., [7]). In general, arsenate is strongly sorbed by most mineral constituents of the sediments, while arsenite exhibits a limited binding, with the exception of iron (hydr)oxides, for which arsenite presents higher affinity than arsenate (e.g., [59]). This limited sorption makes arsenite a more mobile oxyanion [7,24].

Even though less relevant than the aforementioned minerals, other sediment components can also contribute to arsenic sorption. Arsenate is retained by carbonates, either by adsorption or by co-precipitation mechanisms (e.g., [60]). Arsenic may also bind to organic matter in sediments, and amine groups are considered the primary functional group responsible for arsenic retention (e.g., [61]). Arsenic may also bond indirectly to organic groups by bridging with aluminum (Al), iron (Fe), and manganese (Mn) (e.g., [62]). In microorganisms, arsenic retention is a pH dependent electrostatic interaction involving hydroxyl, amide, and amino groups on the variable charge surface of their cells [63,64,65]. 

Environmental conditions affect arsenic adsorption and desorption. Alkalinity (pH >8.5) causes desorption or limited adsorption [66,67,68]. It has been observed that the adsorption of arsenic, like that of phosphate, decreases as the pH increases [69], although the release mechanisms at different pH depend on the experimental conditions, including Eh and the relative concentration of sorbents and the mineralogy and provenance of the sediment samples [66,67,68]. Specifically, in a study [70], it was observed that the percentage of arsenic released from sediments was 10 to 45 times higher at pH 10 than at pH 4. Moreover, leaching of arsenic at alkaline pH was accompanied by the release of Fe, Al, and organic matter, suggesting the association of arsenic with these components that are potentially important sinks for arsenic in sediments. 

Competitive ion displacement may also cause the release of arsenic from sediments to the aqueous phase. Phosphate, carbonate, and bicarbonate ions may inhibit arsenic adsorption and increase arsenic release from mineral surfaces by competing for sorption sites (e.g., [19]). Arsenic release by phosphate is of particular concern [38,59,71,72], since phosphate and arsenate strongly compete for sorption sites, thereby making arsenate more mobile under conditions of phosphate abundance (e.g., [45]). Competition is due to the similarities of phosphate and arsenate molecules, as both form oxyanions, have similar p*Ka* values, and exhibit similar aqueous speciation as a function of pH. In the literature, the mobilization of arsenic by phosphate from sediments has been widely reported by Stollenwerk et al. (2007), Rubinos et al. (2010, 2011), Kaplan et al. (2004) and Bauer et al. (2006) [55,70,73,74,75], among others. The introduction of waters containing high concentrations of phosphate (such as wastewaters or runoff with fertilizers) can cause displacement of arsenic from sorption sites in sediments [76,77,78,79,80]. Competitive effects are also shown by silicate anions (e.g., [81]) and organic matter [82,83,84,85]. Anions such as Cl^−^(chloride), SO_4_^2−^, and NO_3_^−^ (nitrate) have less impact on arsenic retention [86].

## 5. Microbial Biofilms in Freshwater Systems

For many years, the study of arsenic cycling was focused on chemical and physical processes, but there is also strong evidence of the important role that microorganisms play in the arsenic cycle (e.g., [24,29]). In freshwater systems, these microorganisms may occur as complex and structured benthic communities living closely together in a matrix composed of extracellular polymeric substances (EPS matrix), mainly produced by microalgae (diatoms, in particular). These structured benthic communities are generally called biofilms, consisting of a mix of photoautotrophs (including green algae, diatoms, and cyanobacteria) and heterotrophs (including bacteria, fungi, and protozoa) [87,88,89]. 

**Figure 2:**
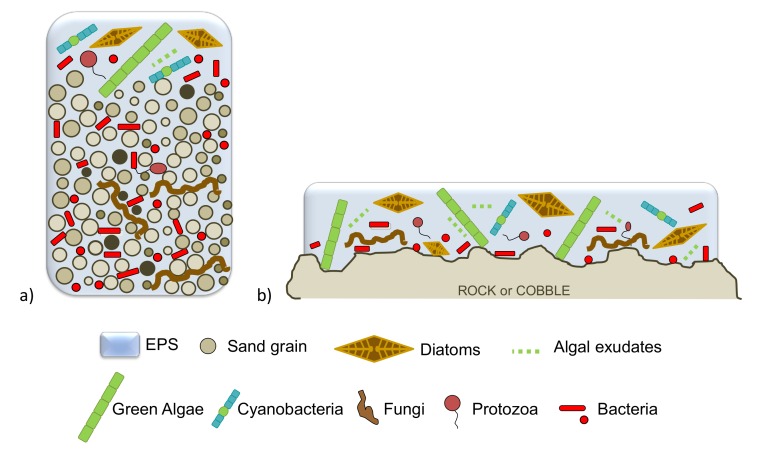
Freshwater biofilm types regarding their growth on (**a**) sand, named epipsammon, or on (**b**) inorganic hard substrates like rock, named epilithon or periphyton EPS: extracellular polymeric substances. Figure adapted from Mora-Gómez et al. (2016) [88].

Aquatic biofilms have a large variability in structure and composition depending on the type of substratum where they develop and the environment in which they are living (e.g., [90]). Biofilms attached to the particles of sandy sediments are referred to as epipsammon or epipsammic biofilms (Figure 2a); while those attached to natural inorganic-hard surfaces, such as rock, gravel, and cobble, are referred to as epilithic biofilms, epilithon, or more generally, periphyton (e.g., [88]; Figure 2b). Epilithic biofilms develop a more complex structure with a higher microalgal biomass, and they are more independent of seasonal fluctuations, compared to epipsammic biofilms (e.g., [91,92]).

In biofilms, several microniches corresponding to different physiological requirements coexist, explaining why opposing redox processes can occur simultaneously in spatially separated locations of the same biofilm environment (e.g., [31]). Moreover, biofilms are complex sets of communities that may experience large diel variations in oxygen concentration as a result of daytime net photosynthesis and nighttime net respiration (e.g., [93]). Therefore, we may consider biofilms as a community and also as an environment for microorganisms. Such an environment may provide niches for arsenate reducers and arsenite oxidizers, so allowing arsenic oxidation and reduction to take place in biofilms (e.g., [31,93]).

Cations, anions, and apolar compounds in water may adhere to and be adsorbed by the EPS via physical or chemical mechanisms. Furthermore, as mentioned above, dissolved organic matter and other dissolved ions (like phosphate) can share similar properties as dissolved arsenic and can compete for binding sites on biofilms [94]. Consequently, biofilms can show the effects of water chemistry. This is the reason why they have been widely applied in biomonitoring of rivers and lakes, along with the physical and geomorphological characteristics. Further details of biosorption are discussed in Section 6. Among biofilm components, diatoms (microscopic and unicellular “brown algae”) are extensively used as reliable environmental bioindicators of both toxicity (e.g., [95,96,97]) and recovery in fluvial ecosystems (e.g., [98]). 

From now on, in the text, we will consider as “microalgae” all aerobic-photoautotrophic organisms, including eukaryotes and cyanobacteria. We will use “prokaryotes” to refer to archaea and bacteria (except cyanobacteria), including all heterotrophic and chemolithoautotrophic microorganisms, as well as anaerobic-photoautotrophic bacteria (e.g., purple bacteria). 

## 6. Arsenic Biosorption Mediated by Biofilms

The efficacy of a biofilm in removal of arsenic from the water column depends on a variety of factors, including the species composition of the biofilm, the relative expression of the various functional groups, and their respective affinity for arsenic; also, the physicochemical conditions of the environment, including the relative concentration of compounds that behave similarly to arsenic and can either bind directly to arsenic or compete for binding sites on mineral and biofilm surfaces [94,99]. Several types of functional groups on the biofilm cell surfaces, such as hydroxyl, carboxyl, carbonyl, sulfhydryl, phosphate, and amino groups, are responsible for superficial adsorption of arsenic [3,63,64,65,100]. The type of functional groups present in the biofilm and associated organic material can facilitate either anion exchange (in the case of hydroxyl, carboxyl, phosphate, and sulfate groups) and cation exchange (in the case of amino groups) [101]. 

Copper is an example of an element that can either compete with arsenic for adsorption sites on either the functional groups of biofilms or the mineral substrate or it can form a precipitate with arsenic [102]. At pH values from 4–9, copper can complex and then precipitate with arsenic [103] or it can compete with arsenic for adsorption sites on the biofilm or substrate surface [104]. Many other dissolved materials, including both organic and inorganic matter, can complex with arsenic and share similar sources, so understanding the chemical composition of water is essential for interpreting arsenic dynamics in a specific system [105,106].

Adsorption is a fast and reversible process and plays a significant role in environmental arsenic detoxification by a wide variety of prokaryote and microalgae species. Interestingly, microalgae may adsorb up to 79% of total arsenic in water, depending on both the absolute concentration and concentration relative to other elements that either bind arsenic or compete with arsenic for sorption sites [103,104,107]. Thus, biofilms are effective in bioremediation efforts (e.g., [3,100]). As mentioned above, adsorption of arsenic is influenced by pH. The highest content of arsenite and arsenate sorbed to microorganisms is typically observed at pH values between 7 and 7.5 (e.g., [31]).

In addition to extracellular sorption, microalgae and prokaryotes can also take arsenic into their cells, but they do not have a dedicated arsenic uptake system (e.g., [30]). Instead, arsenic transport into the cells is achieved via existing systems, like phosphate transport for arsenate and aquaglyceroporins (AQP) for arsenite. The chemical similarity between PO_4_^3-^ and AsO_4_^3-^ suggests that these anions may compete for the phosphate^-^ uptake system (e.g., [3]). The concentration of phosphorus (P) both in the medium and in the cell can mediate the arsenic uptake and speciation in microorganisms (e.g., [108,109]). Some experimental studies have shown that a large quantity of arsenate may be taken up by starved freshwater microalgae because of phosphate deficiency (e.g., [110]); while increasing phosphate in the medium would lead to decreased uptake of arsenate and resulting toxicity [108,110]. Concerning the uptake of arsenite, membrane hexose permeases and AQP were detected to be the transporting systems at physiological pH (~pH 7.4) since, under these conditions, non-charged As(OH)_3_ dominates as the arsenite oxyanionic form [3,30,111]. 

Once inside the cell, arsenic may be metabolized, which involves different arsenic transformations (speciation) that will be explained later on (Section 8). Finally, arsenic species may then be detoxified and excreted or remain sequestered in the cell (e.g., [31]). Arsenic speciation can also happen extracellularly, in the case of only cell surface adsorption.

## 7. Effects of Arsenic Toxicity in Microorganisms and in Trophic Interactions 

The response of microorganisms to arsenic is known to result in various biological effects, including oxidative stress, DNA damage, alteration of the synthesis of EPS, and ultimately, affecting the formation of biofilm (e.g., [112]). In addition, different arsenic species have different modes of toxic action to organisms (e.g., [113]). Arsenite inhibits the production of enzymatic scavengers of oxidative damage such as glutathione, used for the production of phytochelatins (PCs), which are important molecules for the detoxification of numerous metals in phytoplankton and plants (e.g., [113]). Consequently, arsenite toxicity in the cell is due to the synthesis of reactive oxygen species (ROS), which results in the disruption of the membrane and cellular death (e.g., [3]). Regarding arsenate, its toxicity is due to its structural similarity to inorganic phosphate, which leads to oxidative stress and cell division inhibition [3,108]. However, studies conducted with algal cultures demonstrated that increases in P in the culture media can significantly decrease the toxicity of arsenite and arsenate (e.g., [3]). For instance, increasing the phosphate concentration from 1 μM to 10 μM (e.g., [110]) may decrease the growth inhibition of freshwater microalgae by arsenic and enhance the tolerance to arsenate [108,110], but not under excess of phosphate in the medium (e.g., 175 μM in Guo et al. 2011 [110]). In the literature, there are notable differences concerning which P concentrations are considered as P-limited or non-P limited conditions (e.g., excess P is 20 μM for Hellweger et al. 2003 [114]; whereas it is 175 μM for Guo et al. 2011 [110]). Moreover, in addition to the environment and in the culture medium, small variations of intracellular phosphate concentrations could significantly change the toxicity of arsenate in microalgae [109,114]. Therefore, in addition to the analysis of the total arsenic content in the cell, prediction of arsenic toxicity in microalgae may be improved by analyzing the cellular As:P ratio [108,109,115]. 

As an example, some arsenic ecotoxicity data for biofilms (periphyton) and diatoms are shown in Table 1, compiled from the Pesticide Action Network (PAN) Pesticide Database [116]. These data were obtained by measuring different toxicity endpoints, including IC_20_ values (concentrations of arsenic that induced 20% inhibition effect relative to controls), LOEL or LOEC values (the “Lowest Observed Effect Level or Concentration”, or the lowest concentration at which adverse effects are observed), and NOEL or NOEC values (“No Observed Effect Level or Concentration”, or the concentration below which no adverse effects are observed). Mean arsenic toxicity values for periphyton range from 15 to 59.9 µg As L^−1^; while specifically for diatoms, the values may range from 25 to 150 µg As L^−1^. Toxicity values of different arsenic species (arsenate and arsenite) for algae (Table 1) were gathered from the ECOTOXicology knowledge base [117] by Tuulaikhuu (2016) [118], including the LC_50_ value (“Letal Concentration 50”, that is, the concentration of a substance that causes 50% of mortality) set at 79.4 mg As^V^ L^−1^ and the NOEC values set at 1.19 mg As^V^ L^−1^ and 8.59 mg As^III^ L^−1^. Looking at the data extracted from both databases, we detected some differences among which we highlight that most of the data from the ECOTOX database are based on monospecific acute toxicity tests and therefore lack ecological realism, which was also indicated by [118]. Chronic exposure investigations performed in broader ecological scales are crucial to understand the consequences of arsenic exposure on the structure and function of aquatic communities and ecosystems (e.g., [118]).

Changes in the structure and function of biofilms exposed to arsenate have been demonstrated (e.g., [115,118,119]). It was also detected that environmental phosphate concentration may mitigate arsenate uptake and the resulting toxicity in biofilms. For instance, epilithic biofilm communities starved of phosphorus, and chronically (four weeks) exposed to 130 µg As^V^ L^−1^, showed affected structural and functional parameters, inhibition of algal growth and photosynthetic capacity, and changes in the algal community composition, which reduced their ability to retain phosphorus and led to accumulating more arsenic into the cells [115]. Arsenic tolerance was only induced by the combination of arsenate and high phosphorus treatments indicating that tolerance induction may be an ATP-dependent mechanism. In addition, it was also shown that arsenic retention was reduced when phosphate was not a limiting factor [115]. A shorter exposure experiment (130 μg As^V^ L^−1^ over 13 days) carried out under low phosphate concentrations showed negative effects on microalgae structure and function, impeding their growth and becoming a less phototrophic biofilm [120]. Arsenic even impeded the algal succession process during biofilm formation, driving changes in algal community composition. In diatoms in particular, species sensitive to arsenic and large-sized individuals decreased, likely resulting in an increase in overall community tolerance to the metalloid [120].

**Figure 3:**
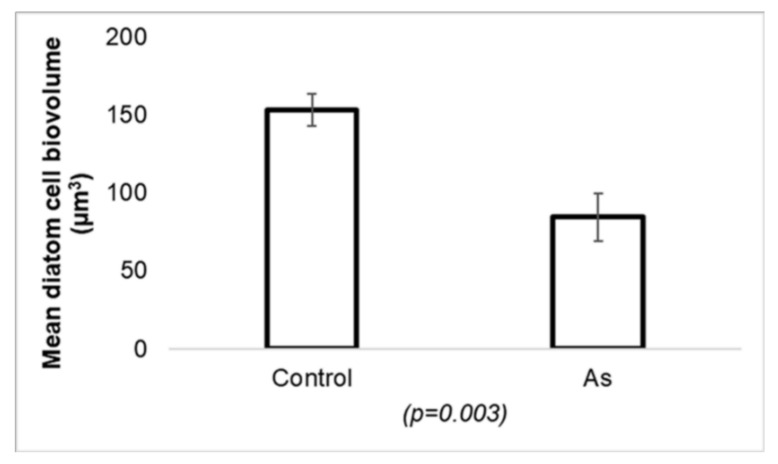
Graph representing a significant change (*p* = 0.003) in the average diatom cell biovolume (μm^3^) when comparing species that were not exposed to arsenic (control) with those exposed to 130 µg As L^−1^ (As). Figure modified from Barral-Fraga et al. (2016) [120].

Diatoms are usually the most represented autotrophic group in aquatic biofilms (e.g., [95,96,121,122,123], which makes them good biological indicators of water quality (e.g., [97,98,124,125,126]). Diatom communities are likely to accumulate significant quantities of metals (e.g., [127,128,129,130]). Numerous works have described the toxicity mechanisms of metals in diatoms. However, the toxicity caused by some metalloids, arsenic in particular, has not been extensively studied, although similar effects may be expected. Several studies described diatoms as highly sensitive to toxicants compared to other aerobic photosynthetic microorganisms (e.g., [131,132,133]). However, another study found that diatoms adapt arsenic tolerance under acute arsenic exposure (e.g., [120]) and become more resistant to this metalloid than other microalgal groups (green algae and cyanobacteria). This resistance had a cost: a clear decrease of real cell size or cell biovolume (Figure 3) and a slight loss of species richness (*S*) [115,120]. A decrease in diatom cell size following exposure to high metal concentrations may be caused by a higher cellular division rate during vegetative reproduction, typical under stressed conditions [43,123,134,135,136,137].

Arsenic levels in natural systems are often well below those that cause mortality in higher organisms such as fish, but even low concentrations may impede normal functioning. In a laboratory study [138], fish exposed to dissolved arsenic (130 μg L^−1^) were more aggressive and had higher arsenic tissue content (around 600 μg g^−1^ or ppb) than those not exposed to the metalloid. However, it is worth noting that the highest arsenic bioaccumulation (almost 800 μg g^−1^) and strongest aggression in fish were detected when including biofilm in the experiment, as an effect from the interaction between fish and biofilm. This study highlights the importance of including multiple trophic levels, from microorganisms to higher organisms, in ecotoxicological studies.

Biofilms constitute important sources of energy for invertebrates and herbivorous fish (e.g., [139]). Moreover, they are not only a site for biotransformation, but also a site for the transfer of chemicals to higher organisms (e.g., [140]). Direct effects of toxicants on the most sensitive community (e.g., microalgae and/or prokaryotes) may trigger indirect effects on the rest of the community and also affect higher organisms of the food web, since all of them are closely related through biological interactions (e.g., [140,141]). Under natural conditions, the interaction between biofilms and fish is also related to nutrient cycling since fish have an important role in releasing nutrients, and biofilms may actively take them up. However, arsenic may change the nutrient dynamics and influence the whole ecosystem. For instance, a study [142] assessed the effects of 120 µg As^V^ L^−1^ on periphyton, epipsammon, and fish under P-limiting conditions (around 6 µg L^−1^). Total dissolved arsenic concentration decreased exponentially to 28 µg As L^−1^ during the two month test, mostly due to adsorption to the sediment, but with a small amount accumulated in the periphyton. Almost all nutrients were also retained in the epipsammon. Arsenic effects on fish were decreased in the presence of biofilms at the beginning of the exposure, but not later on, since the arsenic-affected biofilm had lost its role in arsenic detoxification some days after (around Day 30 for cyanobacteria and diatoms). After longer exposure (56 days), arsenic reduced the total biomass of biofilm and its potential ability to use organic phosphorus (i.e., phosphatase activity), inhibiting algal growth, especially that of diatoms, and making the epipsammon less autotrophic. 

In conclusion, damaged biofilms may influence the fitness of higher organisms, such as fish, and overall ecosystem function [100]. Moreover, communities of microorganisms in biofilms also contribute to nutrient cycling, and arsenic may influence this ecosystem service.

## 8. Changes in Arsenic Toxicity through Microbiological Biospeciation

Despite the high toxicity of the two inorganic forms of arsenic (arsenate and arsenite), some microorganisms can play a crucial role in arsenic biogeochemistry and toxicity by transforming those forms during metabolic processes [143]. Arsenic metabolism may be affected by factors such as growth medium, arsenic species and concentration, pH, temperature, Eh, exposure duration, and light intensity and photoperiod (e.g., [3]). Moreover, microorganisms may transform arsenic into less or more toxic forms depending on the concentration of nutrients like phosphate. A huge diversity of arsenic species may be found in experiments with algae or biofilms when they are exposed to different P concentrations, showing the complexity of the role of P in arsenic biospeciation. For instance, in a field study [144], the role of total P on the biotransformation of arsenic was tested and suggested that the increase of arsenite mobility in water by microorganisms was enhanced under low phosphate conditions, after arsenate was rapidly taken up by microalgae via phosphate transporters and then reduced to arsenite. 

Production of methylarsenicals was generally observed as a detoxification process and was detected inside of microalgal cells and fluvial biofilms, especially under eutrophic conditions (e.g., [119,144]). The remarkable production of methylarsenicals by freshwater green algae at high P concentrations was also demonstrated by Baker et al. (2016) [145], calling into question the conclusion made by some researchers that low P concentration is essential in the production of these organoarsenicals (e.g., [114,146,147]). Consequently, more studies regarding the influence of P on the biogeochemical cycle of arsenic species in aquatic environment are required, particularly in assessing the role of biofilms.

**Figure 4:**
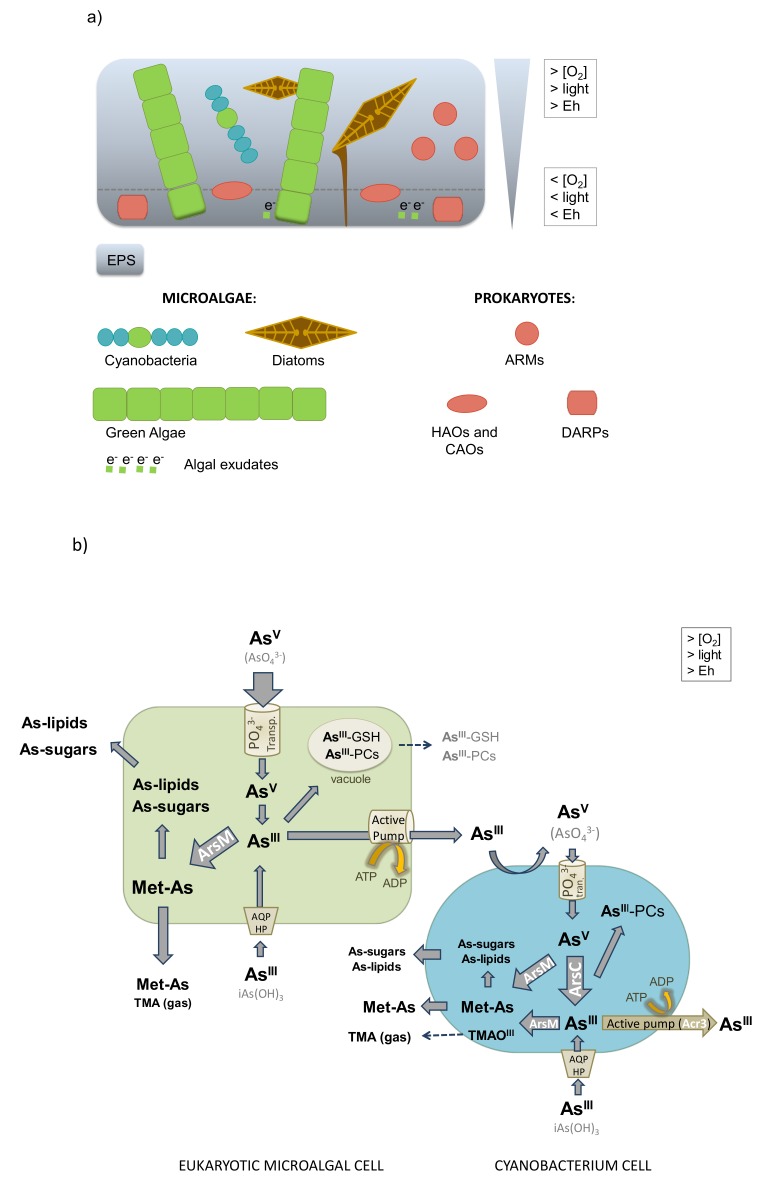

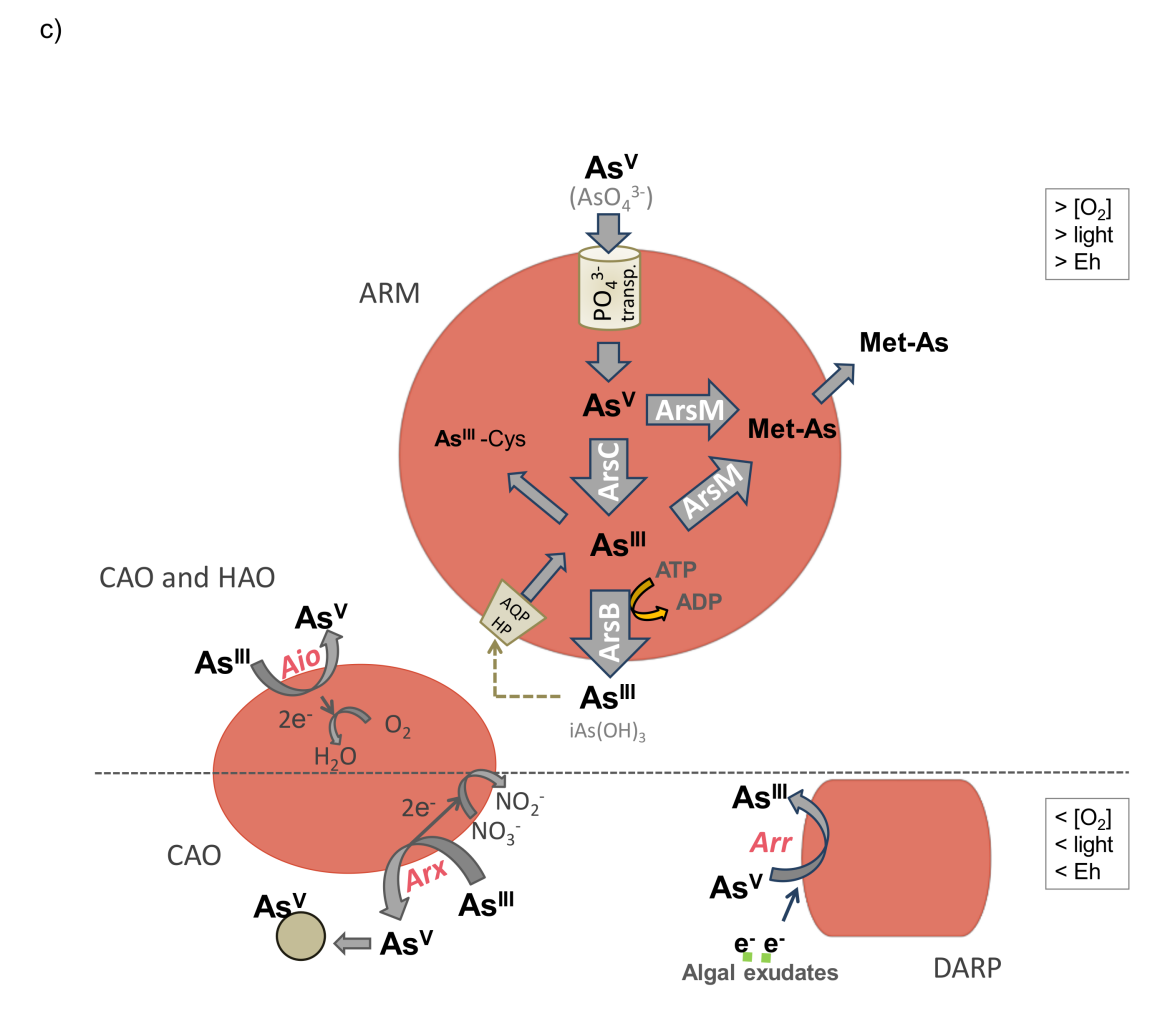
Arsenic speciation by prokaryotes and microalgae, together being part of biofilms. Biofilm redox profile zonation in depth and consequent location of different microorganisms, mainly microalgae and prokaryotes (**a**). Speciation in freshwater biofilms: main speciation processes in cyanobacterium and eukaryotic microalgae cells (green algae and diatoms) (**b**), and in aerobic and anaerobic prokaryotes (**c**). ARMs: arsenate-resistant microorganisms. HAOs: heterotrophic arsenite oxidizers. CAOs: chemolithoautotrophic arsenite oxidizers. DARPs: dissimilatory arsenate-respiring prokaryotes. Transp.: transporters. AQP: aquaglyceroporins. HP: hexose permeases. GSH: glutathione. PCs: phytochelatins. Cys: cysteine residues in enzymes. See the main text for details.

The role of microorganisms in arsenic biochemistry is generally explained separately for prokaryotes and microalgae in most cases (e.g., [3,30,31,143,148]). Here, we review the general information of both kinds of microorganisms together being part of biofilms (Figure 4a) to better understand how they could interact in such structured communities to affect arsenic speciation in freshwater ecosystems. Finally, some figures were designed to illustrate and simplify the most known pathways of arsenic speciation in microorganisms (Figure 4b,c). 

### 8.1. Arsenite Oxidation

Oxidation of arsenite to arsenate by microorganisms has an important impact on arsenic availability because it decreases arsenic mobility in the environment due to the higher affinity of arsenate than arsenite for mineral surfaces. Moreover, it is a detoxification process because arsenate is typically less toxic than arsenite to prokaryotes, higher organisms such as fish, and most organisms [3,30,113,149]. Microbial oxidation can be faster than chemical oxidation of arsenite to arsenate (e.g., [150,151]). In microalgae, biotransformation of arsenite to arsenate has received little attention, but has been observed in the cell surface of some acidophilic red algae and some cyanobacteria (see Figure 4a), especially under increased P levels in the medium [152,153]. Furthermore, arsenite oxidation is usually observed on the outer membrane of bacteria cells, including anaerobic-photosynthetic purple bacteria (e.g., [154]). In prokaryotes, arsenite oxidation has been particularly well studied in the chemolithoautotrophic arsenite oxidizers (CAOs) and heterotrophic arsenite oxidizers (HAOs) (Figure 4c). All known CAOs gain energy from the oxidation of arsenite to arsenate under aerobic and anoxic conditions; while HAOs convert arsenite into the less toxic arsenate form only under aerobic conditions [29,30,31,113,151]. Under aerobic conditions, both HAOs and CAOs use the key enzyme arsenite oxidase, named Aio, resulting in the production of ATP and the reduction of oxygen to water. Conversely, the anaerobic arsenite oxidation by CAOs (using nitrate instead of oxygen as the electron acceptor) is via the arsenite oxidase Arx enzyme, which is less well characterized (e.g., [151,155]).

### 8.2. Arsenate Reduction

Reduction of arsenate to arsenite leads to an increase in arsenic mobility and toxicity in the natural environment (e.g., [3]). It has been commonly observed in prokaryotes and microalgae [24,113], both extra- (anaerobic process) and intra-cellularly (aerobic process). 

Microorganisms that reduce arsenate to arsenite under aerobic conditions (that is, via intracellular processes) and as a means of resistance are called arsenate-resistant microorganisms (ARMs, referring especially to prokaryotes; see Figure 4c). These microorganisms actively take up the toxic arsenate through the phosphate uptake system and can then expel the metalloid from the cell, after reducing it to arsenite to facilitate its export [3,29,113]. The best-studied resistance mechanism is the Ars (arsenic resistance system) operon, including a cytoplasmic arsenate reductase enzyme (ArsC), that reduces arsenate to the far more soluble arsenite, and an arsenite efflux pump (ArsB) that excretes arsenite out of the cell. Many studies support this biotransformation model as a mechanism of survival in high arsenate environments (e.g., [29] in microalgae (Figure 4b), including cyanobacteria [156,157], and in aerobic prokaryotes (Figure 4c)) [3,108,151,155]. If, instead of arsenate, the aqueous arsenite directly enters the cell, it can be pumped straight out (e.g., [151]) or, in microalgae, easily excreted from the cells through previous sequestration as stable complexes with glutathione (GSH) or phytochelatins into the vacuole (e.g., [3,113]. 

In turn, microorganisms that reduce arsenate to arsenite under anaerobic conditions (that is, via extracellular processes) are anaerobic prokaryotes called dissimilatory arsenate-reducing or arsenate-respiring prokaryotes (DARPs), opportunists capable of respiratory growth on a wide diversity of electron donors (e.g., [29,93]). For instance, in biofilms, algal exudates are an important source of organic matter and would provide an abundant supply of electron donors to these microorganisms (e.g., [93]). This anaerobic respiration can be done in the bottom layer of biofilms (Figure 4a), caused by the microbial mats themselves due to microbial respiration and the onset of anoxia (e.g., [24]). In subsurface water aquifers, DARPs can respire arsenate adsorbed to minerals (e.g., ferrihydrite), through an arsenate reductase named Arr (e.g., [112]), resulting in the production and release of arsenite into the aqueous phase (e.g., [24]).

### 8.3. Arsenic Methylation and Demethylation

Methylarsenicals (Met-As) are typically excreted to the aquatic environment by microorganisms, including prokaryotes and microalgae, through reduction of inorganic arsenic species (arsenate and arsenite) and subsequent methylation, a reaction catalyzed by the arsenite methyltransferase (ArsM) enzyme [3,30]. Biomethylation can be a detoxifying process depending on which Met-As species the microorganisms predominantly produce. Arsenic methylation by microalgae has been especially well documented (Figure 4b). 

Arsenate can be methylated to the less toxic organic arsenic species monomethylarsenate (MMA^V^; also named monomethylarsonic acid, MMAA^V^) and to dimethylarsenate (DMA^V^; also named dimethylarsinic acid, DMAA^V^) (e.g., [3,152,155]). In recent years, it has become apparent that methylation of arsenite may not be necessarily a detoxification process, as various metabolic species can be produced that are even more toxic than their arsenite analogs, such as the monomethylarsenite (MMA^III^, or methylarsonous acid, MMAA^III^), and dimethylarsenite (DMA^III^ or dimethylarsinous acid, DMAA^III^). Arsenite methylation can also produce trimethylarsine oxide (TMAO), which seems to be not very noxious for fluvial diatoms (e.g., [158]), and the volatile trimethylarsine (TMA), a gaseous arsine produced mainly by bacteria (e.g., [159]). Biological production of TMA by epipsammic biofilms is minor, as shown by Prieto et al. (2016) [160], and particularly low in cyanobacteria (e.g., [161]). Some studies show that TMA is relatively non-toxic [3,162], but there is also recent evidence suggesting that it can be toxic to mammalian cells even at low concentrations (e.g., [159]). Generally, the toxicity of arsenic species (most to least) for organisms would be MMA^III^ = DMA^III^ > As^III^ > As^V^ > MMA^V^ = DMA^V^ > TMAO^V^ = TMAO^III^ > TMA [3,113]. 

The mechanisms driving biomethylation of inorganic arsenic species by microorganisms are controversial (e.g., [3,31,113]). Various methylation pathways have been proposed. In 1945, the “challenger pathway”, an alternation of reduction and oxidative methylation reactions leading to the formation of trimethylarsine as the end product, provided one possible explanation of arsenic methylation and volatilization, but does not explain the production of arsine, mono-, and dimethylarsine [159]. Currently, it is still difficult to prove which mechanisms occur, and it is most likely that different pathways occur depending on the species of microorganisms and the type of environmental factors (e.g., [159]), such as especially nutritive status (mostly P conditions), light, and temperature (e.g., [113]). High arsenic concentrations and/or long exposure times may be other important factors causing the production and efflux of methylated arsenic species. Furthermore, it can be possible to find Met-As species in natural waters as a consequence of the breakdown of dead cells and the degradation of arsenosugars or arsenolipids from decomposing cells [3,24]. 

Demethylation of a methylarsenic molecule is the chemical process resulting in the removal of a methyl group (CH_3_). Although As-demethylation by microorganisms has been broadly described in the natural environment, little is known about microbial demethylation processes and the involved microbial community (e.g., [31]). It is expected that this biodemethylation may have important implications in the arsenic cycle and in the ecological status of aquatic systems, since it may increase the release of inorganic arsenic species into the water and, consequently, increase its toxicity (e.g., [31]).

### 8.4. Synthesis of Arsenosugars and Arsenolipids

Microorganisms may also synthesize organic arsenosugars (arsenic-containing sugars) and arsenolipids (arsenic-containing lipids). Their biosynthesis has been better studied in marine environments than in freshwaters. Nonetheless, it was detected that some freshwater microalgae (Figure 4b) may biosynthesize arsenosugars after arsenate reduction and posterior methylation (e.g., [162,163]). Arsenosugars are less toxic than their inorganic counterparts and are of special interest because they are widespread in many aquatic organisms (e.g., [19]). The characteristics and effects of arsenolipids are poorly known because of the difficulty isolating and analyzing them correctly (e.g., [3]). However, it has been shown that arsenolipid biosynthesis can be catalyzed by ArsM in a freshwater cyanobacterium, especially under low arsenate concentrations (e.g., [164]). It seems that some arsenolipids could be transformed into more harmful arsenic species in higher organisms (e.g., [165]), which could increase arsenic toxicity in the environment, with potential effects on ecosystem functioning.

## 9. Cases of Arsenic-Impacted Sites: Current Knowledge and Future Research Needs 

In this final section, we present two real cases of arsenic exposure, Pampean Streams (Argentina) and the Anllóns River (Galicia, Spain), two examples that have improved our understanding of arsenic biogeochemistry and toxicity in freshwaters over the last few years, and from which the idea of this review was born. Here, we explore these different case studies of arsenic-impacted fluvial ecosystems as examples of natural and anthropogenic arsenic pollution, and we finish by pointing out some future research topics that would advance the knowledge of arsenic ecotoxicology and biogeochemistry in freshwaters.

### 9.1. Pampean Streams: Effects of Naturally-Occurring Arsenate in Surface Waters 

High natural arsenic concentrations are detected in surface waters of the Pampean streams; this is considered an important health issue, but little is known about its environmental impact. The Pampean plain is a broad part of Argentina (1 × 10^6^ km^2^), and water supply is a huge concern for rural populations [166]. Many studies have focused on Pampean water quality after the detection of arsenic in both groundwater [167,168,169,170] and in surface waters [171,172,173], particularly in lotic environments [174,175]. In studies carried out in the province of Buenos Aires, part of the Pampean region, arsenic levels have been surveyed [174] in 39 Pampean streams, finding values above those recommended for the protection of the aquatic biota (average 114 µg L^−1^). Furthermore, these streams have exhibited a wide range of phosphate concentrations (e.g., [176,177]). 

Arsenic levels in Pampean surface waters have been attributed to the hydrogeology of the streams, fed by an aquifer with high levels of arsenic (0.6 to 4.9 mg L^−1^) originated from Quaternary loess sediments (e.g., [178]) with volcanic glass [179,180], which are mobilized in aerobic or basic waters. Arsenic bioaccumulation has been studied in filamentous algae, biofilms, and fish [172,175,179,180,181]. Arsenic concentration ranges from 2.9 to 33.9 µg g DW^−1^ in filamentous algae, from 14.2 to 214.7 µg g DW^−1^ in freshwater biofilms, and from 1.04 to 3.55 µg g DW^−1^ in fish tissue, indicating that no biomagnification occurs with this metalloid at Pampean streams [182]. However, these values are among the highest reported worldwide for fish (e.g., [183,184,185]). Furthermore, no correlation has been found between arsenic levels in the water column and those accumulated by these organisms, suggesting that its bioavailability depends on factors other than dissolved arsenic concentration, such as co-occurrence with dissolved phosphorus, humic acids, and sediments, all components that may influence arsenic bioavailability [186,187,188]. The influence of dissolved phosphorus on arsenic toxicity has been shown in a study that detected differences in structural and functional parameters in biofilms developed in seven Pampean streams with different arsenic and phosphorus concentrations. Communities grown in streams with high arsenic concentrations have shown low algal growth, but this effect was minimized when phosphorus concentration was high [175]. In this case, it is clear that more research is needed to better understand the bioavailability and biomagnification of arsenic in the organisms living in these peculiar streams. 

### 9.2. The Anllóns River: Polluted Sediments Resulting from Former Mining Activities 

In contrast to the Pampean streams, arsenic concentration is very low in the water of the Anllóns River (Galicia, NW Spain). In this case, arsenic pollution is mainly found in surface and subsurface sediments, and it is attributed to natural geogenic arsenic enrichment exacerbated by mining activities [189]. Arsenopyrite mineralization in hydrothermal quartz veins [190] is associated with gold ore, which was exploited during the Roman Empire and then from 1895 until 1910, with intermittent extractions after that period. Arsenic concentrations in the rocks of the area are usually around 1%, although in mineralized zones with arsenopyrite, they can reach up to 10%. Soils are also enriched in arsenic in the mineralized areas, where concentrations of 4000 mg kg^−1^ have been detected [191]. In a wider study, total arsenic ranging between 2 and 489 mg kg^−1^ was determined in fifty soil samples of C-horizons, covering an area of 50 km^2^ along the Anllóns River [68]. Arsenic leachability using the standard methods [192] and TCLP (Toxicity Characteristic Leaching Procedure) was less than 0.25% of total arsenic. The results of this study suggest that the mobilization of arsenic may be underestimated in short-term water leaching tests and that the environmental conditions favoring arsenic mobilization (presence of phosphate, high pH, low solid:liquid ratio, and long residence time) should be taken into account for a sound evaluation of the transfer risk of arsenic towards aquatic ecosystems.

In the riverbed sediments, concentrations as high as 264 mg As kg^−1^ were detected downstream of the mineralized area to the river mouth [70,189]. Higher values (up to 308 mg kg^−1^) were even found at the estuary and estimated that the Anllóns River exports to its estuary 460 kg y^−1^ of dissolved (<7% as organic) arsenic annually [193]. Geochemical investigations showed that most arsenic in the sediments of the Anllóns River is associated with low-mobility phases [73,189], specifically as bound to Fe-oxide forms and in the residual phase. Nevertheless, as in the soils, this apparent low arsenic mobility may dramatically increase with changes in the environmental conditions such as increasing water:sediment ratios, as those occurring during high-flow resuspension events [70]. Furthermore, as mentioned above, arsenic mobility is strongly dependent on the pH, and it occurs simultaneously with the dissolution of components with which it is associated: oxides and hydroxides of Fe and Al at acidic pH and organic matter at alkaline pH [73]. Arsenic release is also promoted in high ionic strength conditions, as it is characteristic of estuarine environments where the mixture of fresh and marine waters occurs [73]. The release of arsenic is favored by the presence of phosphate, showing high concentrations in some sections of the river [194,195,196], coming from wastewaters discharged into the river course and fertilizers eroded or leached from the soils of the basin. Interestingly, although phosphate favors arsenic release from the Anllóns sediments, it was shown in Microtox^®^ bioassays that it counteracts the acute toxicity of arsenate, but has no effect on the toxicity of arsenite and DMA^V^ [197]. 

**Figure 5:**
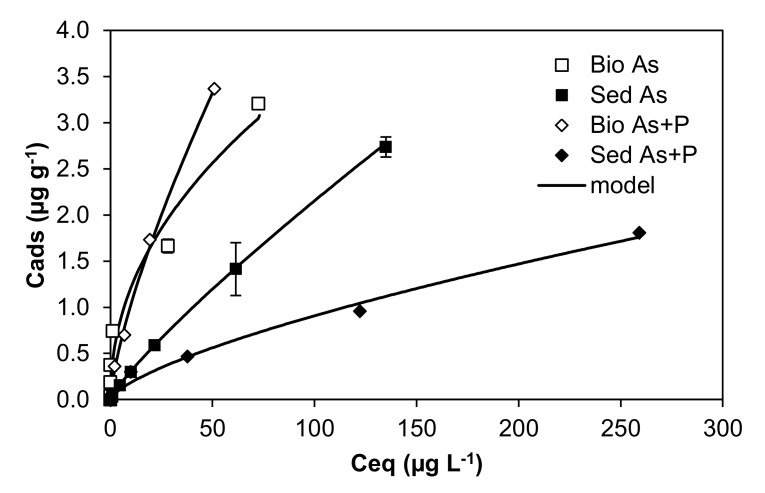
Effect of biofilm and phosphate addition on arsenate retention by sediments from the Anllóns River (Freundlich model). Retention experiments were carried out at pH 5.5, and the arsenic concentrations assayed were 0, 5, 25, 50, 100, 250, and 500 µg L^−1^ prepared in Milli-Q water and 0.01 M CaCl_2_ as the background electrolyte. C_eq_: equilibrium arsenic concentration in solution. C_ads_: adsorbed arsenic concentration by sediments (Sed As and Sed As + P indicate that sediments are exposed to arsenic concentrations and to arsenic and P concentrations at equimolar ratios, respectively) and by sediments covered by biofilm (Bio As and Bio As + P). Figure modified from Prieto et al. (2013) [107].

The microbial communities from the river sediments also affect arsenic biogeochemistry. The riverbed of the Anllóns River is covered by epipsammic biofilms [198], dominated by diatoms [199]. Epipsammic biofilm inocula from this river incubated at a laboratory scale in experimental fluvial channels and bioreactors [200] affected both the mobility and speciation of arsenate in the water column and sediment. Batch adsorption experiments performed with sediments covered by epipsammon revealed that biofilms increased the retention of dissolved arsenic by the Anllóns sediments [107], particularly in the presence of phosphate (Figure 5). This behavior was confirmed in bioreactor experiments, which showed that the biofilms increased the retention of arsenate (up to ~97%) from the water column in comparison with the sediment without biofilm (~70%) [133]. In the presence of the biofilm, there was almost no reduction of arsenate to arsenite in the water column, while the occurrence of organic species such as MMA^V^ and DMA^V^, resulting from biological transformations, was promoted [200]. Most of the arsenic in the biofilm was retained in extracellular compartment (~71%), almost exclusively in the form of arsenate (~99.5%). 

**Figure 6:**
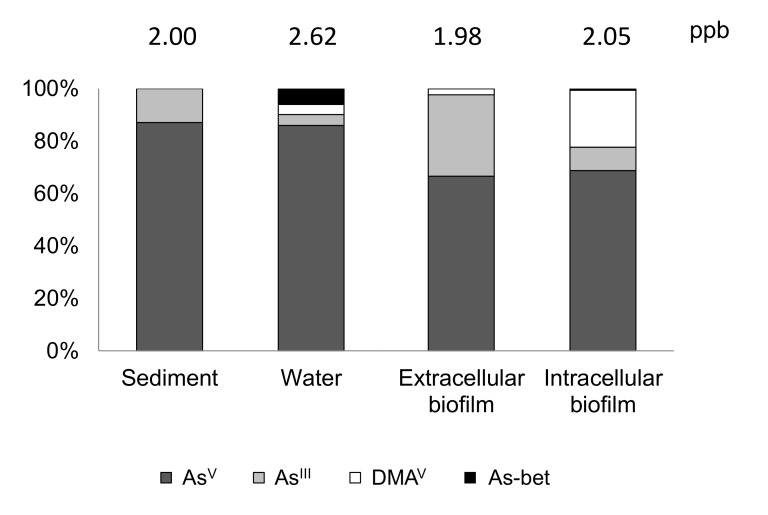
Arsenic speciation in the site located downstream of the mine area in Anllóns River. Histograms show the variability of arsenic speciation profiles in this site depending on the different analyzed compartments: sediment (the easily-extractable arsenic concentration), river water, and biofilm (extracellular and intracellular fractions), based on two sampling occasions over 51 days of exposure. Total mean arsenic concentrations are also shown above each bar expressed in ppb (i.e., in µg As g^−1^ for sediment and biofilm and in µg As L^−1^ for river water). As^V^ = arsenate; As^III^ = arsenite; DMA^V^ = dimethylarsenate; As-Bet = arsenobetaine. Figure modified from Barral-Fraga et al. (2018) [119].

A relevant contribution of benthic biofilms to arsenic biogeochemistry through mobility and speciation processes was also observed in a field experiment performed in the Anllóns River [119], where mobility and speciation are dependent on the epipsammic activity and on inputs of dissolved phosphate into the system. As a consequence, different arsenic species (arsenate, arsenite, and DMA^V^) were finally detected in the studied biofilms (Figure 6). It is worth noting that most arsenic in sediments and water was arsenate. Therefore, the high amount of arsenite detected extracellularly confirmed arsenate reduction by the epilithic biofilms (Figure 6). Moreover, arsenic accumulation was higher in the site with the greatest As:P ratio (proxy for potential arsenic toxicity). In concordance, biofilm growth was reduced, the mortality of bacteria and diatoms was increased (as shown by a higher number of dead bacteria and dead diatom densities), and the nitrogen concentration was lower in this site compared to the less polluted site, located upstream [119].

### 9.3. Future Research Needs 

Overall, these studies performed in Pampean streams and in the Anllóns River highlight the relevance of benthic microbial communities on the behavior of arsenic in freshwaters. These results, as other investigations referenced in this review, demonstrated that both epilithic and epipsammic biofilms play a key role in arsenic retention and biotransformation and that phosphate modulates the toxicity and mobility of arsenic. We know that freshwater biofilms may methylate and detoxify arsenic, but to what extent they do this is not clear, nor are the distinctive roles that microalgae and prokaryotes play (e.g., [112]) or the conditions that trigger arsenic mobilization, biotransformation, and resulting toxicity. 

Arsenic speciation may experience great spatial and temporal changes. Taking into consideration that epilithic biofilms can reduce and methylate arsenic, it is very important to understand if algal metabolism influences arsenic speciation in the water column, therefore affecting arsenic toxicity to other organisms. For example, biofilm metabolism can change water pH and oxygen concentration, and it is well documented that arsenic sorption to Fe oxides is weak at high pH (e.g., [201]) and that arsenic speciation depends on oxygenation. Advanced analytical procedures capable of quantifying arsenic speciation in water, sediment, and biota are urgently needed. The fact that arsenite is quickly oxidized to arsenate in oxygenated conditions could result in arsenite underestimation (e.g., [28,202]). In addition, many determination techniques of arsenic species do not account for the presence of organoarsenicals. Therefore, it is possible that organic arsenic species, if present, may contribute to the total “inorganic arsenic” measurement by some in situ techniques such as some kind of diffusion gradient in thin film (DGT) devices (e.g., [202]). Furthermore, a definitive mechanism to explain the arsenic bio-volatilization process is also needed (e.g., [159]). Future research should focus on the application of effective approaches for the investigation of arsenic speciation, especially in highly productive areas.

The role of phosphate on the cycle of arsenic by microorganisms is a controversial issue, and we lack mechanistic details about the effects of phosphorus on arsenic biogeochemistry, especially regarding microbial processes (uptake, speciation, and excretion). Nutrient availability has a strong influence on arsenic toxicity to freshwater algae and biofilms, which may retain arsenic and transform it into more or less toxic forms depending on phosphate conditions, as well as influence of the toxicity on other higher organisms like fish. Differences in phosphorus availability may explain why the presence of biofilms increased arsenic toxicity to fish in some laboratory experiments [138], but decreased it in those performed under lower phosphorus concentration (e.g., [203]). However, it is still uncertain how phosphate influences the uptake, retention, and transformation of arsenic species in microalgae or biofilms. More detailed studies are needed to solve these uncertainties. Indeed, considering the stoichiometry of phosphorus in relation to other elements like nitrogen (N), which is intimately linked with phosphorus, recent research has shown that uptake of arsenic by microbial biofilms is driven by N:P, not just phosphorus alone [204], and more investigations should also move along this line. It is also crucial to perform new experiments to be able to conclude under which conditions biofilms protect fish and other aquatic organisms from arsenic toxicity. To understand this challenging issue, it will be necessary to explore a larger range of phosphate and arsenic concentrations and carefully consider the arsenic speciation.

Arsenic bioaccumulation in biofilms is relatively high in comparison with other aquatic organisms [205]. However, the role that microorganisms play in arsenic toxicity to higher trophic levels has been poorly addressed. A huge amount of genomic sequences relevant to arsenic cycling is published in databases, including a complete characterization of several bacteria metabolizing arsenic (e.g., [143,206,207]). In this respect, metagenomic approaches may contribute to elucidating these questions (e.g., [112,208,209]). Moreover, since aquatic organisms are chronically exposed to high arsenic concentrations in aquatic systems like the Pampean streams, microbial communities are likely adapted to arsenic. Further, molecular biotechnology studies will enhance our understanding of arsenic resistance mechanisms, for instance through the study of the *“ars”* genes (e.g., [143]). Research may also focus on the function of phytochelatin synthase (pcs) in arsenic toxicity management [3,210]. It is important to highlight that the study of genes of resistance in microbial ecotoxicology is relatively recent, especially in phototrophic organisms (e.g., [211]). 

On top of that, little is known about the impact of human activities such as the discharge of high concentrations of organic matter and/or nutrients in arsenic speciation and mobility in freshwater systems, such as the Anllóns River. Organic matter may play an important role. It is known that humic substances can bind to arsenic, making it unavailable for organisms (e.g., [187]). Arsenic binding to humic acids depends on pH and type of humic acid (e.g., [186]), but the influence that organic matter has on arsenic toxicity to the biofilm community needs more investigation.

Finally, the large set of effects reported on arsenic-exposed biofilms such as the reduction of biomass and algal growth (especially diatoms), the reduction of the proportion of autotrophic vs. heterotrophic organisms, or the lowering nutrient cycling and nitrogen content brings into question the arsenic concentration thresholds established by the U.S. EPA [11] for freshwater systems. The toxicity results described in this review were observed under arsenic concentrations of 130 μg L^−1^ and lower. We conclude that recent studies do not support either the criterion maximum concentration of 340 μg L^−1^ (acute exposure), proposed by the U.S. EPA [11] in freshwaters, or the criterion continuous concentration (chronic exposure) of 150 μg L^−1^, and we suggest that these thresholds should be updated. It is also important to note that arsenic exposure thresholds for human health (10 μg L^−1^, [13,14]) are 15 times lower than those established for environmental health.

## 10. Conclusions

The intention of this review was to describe our current understanding of arsenic biogeochemistry in freshwaters. Using both novel case studies and previously published studies, we highlighted the effects of biofilms to arsenic toxicity and the key role that biofilms play in arsenic biogeochemistry causing potential changes to the ecological status of fluvial systems. Finally, we highlight areas of research that are still sparse and would benefit from additional investigations. Specific areas include better understanding the potential of biofilms to detoxify, transfer to higher trophic levels, and alter arsenic’s speciation in freshwaters, better characterizing arsenic resistance, greater mechanistic detail about the interaction between arsenic and other elements or compounds, and a reevaluation of current contamination thresholds.

## Figures and Tables

**Table 1:** Mean arsenic toxicity values for different exposed organisms (biofilm, algae, and diatoms), according to the PAN and ECOTOX databases.

Database	As-Exposed Organisms	Mean Toxic Dose	Concentration Units	Toxicity Endpoint	Effect Measurement
PAN	Biofilms (Periphyton)	37.5	µg As L^−1^	IC_20_	Carbon content
		59.9			Nitrogen content
		44.9			Photosynthesis
		30			Photosynthesis
		22.5			Photosynthesis
		22.5			Biomass
		15			Diversity
	Diatoms	60	µg As L^−1^	NR	General biochemistry
		150			General biochemistry
		25			General biochemistry
		1.5	pg cell^−1^	LOEC	Abundance
		4.5		NOEC	Abundance
ECOTOX	Algae	79.4	mg As^V^ L^−1^	LC_50_	Abundance
		1.19		NOEC	Biomass
		8.59	mg As^III^ L^−1^	NOEC	Biomass

NR: not reported. LOEC, lowest observed effect concentration; NOEC, no observed effect concentration.
